# Successful palliative resection of giant epimyocardial lymphatic malformation with 14 years of follow-up: a case report

**DOI:** 10.1186/s12872-023-03449-8

**Published:** 2023-08-23

**Authors:** Krista Heliö, Sini Weckström, Sari Kivistö, Jouko Lohi, Tiina Heliö

**Affiliations:** 1 Heart and Lung Center, Helsinki University Hospital and University of Helsinki, Helsinki, Finland; 2 Department of Radiology, University of Helsinki and Helsinki University Hospital, Helsinki, Finland; 3 Department of Pathology, HUSLAB, University of Helsinki and Helsinki University Hospital, Helsinki, Finland

**Keywords:** Benign heart tumors, Lymphatic malformation, Lymphangioma, Cardiac surgery, Case report

## Abstract

**Background:**

Primary tumors of the heart are a rare phenomenon. Lymphatic malformations are congenital anomalies of the lymphatic system that tend to grow progressively. Lymphatic malformations are typically found in the cervical and axillary regions and found on pediatric patients. We report a 40-year-old woman with giant epimyocardial lymphatic malformation.

**Case presentation:**

A 40-year-old woman was assessed due to suspected traumatic cardiac tamponade. Computed tomography of the heart and cardiac magnetic resonance imaging were compatible with either a large pericardial hemangioma or angiosarcoma. The tumor infiltrated deeply into the myocardium and could only be partially resected. Histopathological diagnosis was a cardiac lymphatic malformation with micro- and macrocystic components. The patient has remained asymptomatic for fourteen years after the surgery. In the latest follow-up, her left ventricular function had remained normal and the maximum thickness of the residual tumor had regressed.

**Conclusions:**

Even when a complete removal of a cardiac lymphatic malformation is not possible, a debulking procedure can yield a good long-term result.

## Background

Primary tumors of the heart are a rare phenomenon, and most of them are benign. Secondary tumors of the heart are more common, and in some studies the prevalence has been 20 times greater when compared to primary tumors [1]. Lymphatic malformations are congenital anomalies of the lymphatic system that tend to grow progressively. Lymphatic malformations are typically found in the cervical and axillary regions, although rarer, few cases of cardiac lymphatic malformations have been reported as well [2, 3].

We present a 40-year-old woman with giant epimyocardial lymphatic malformation. This case study makes an important contribution by reporting the results of a long-term post-operative follow-up.

## Case presentation

A 40-year-old woman was admitted urgently to the emergency department of Helsinki University Hospital, Finland, with a suspected traumatic cardiac tamponade. She had attended an outdoor concert where the crowd pushed her against a fence causing an injury to the head and the left side of the chest. Apart from being operated for a cavernous hemangioma in her temporal lobe eleven years earlier, her past medical history was unremarkable, and she did not have any regular medication. She denied any cardiac symptoms and declared to have a normal exercise tolerance.

**Fig. 1:**
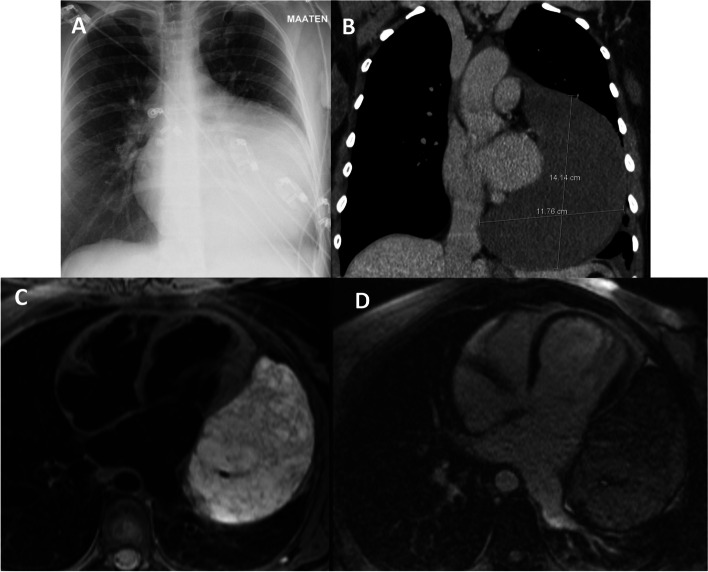
Imaging findings. **A** Chest X-ray showed an enlarged cardiac shadow without pneumothorax. **B** 14 cm diameter cystic pericardial tumor without calcifications, fat or contrast enhancement in CT. Both coronary and CT angiography demonstrated feeding arteries to the tumour from distal branches of LCX and RCA and venous drainage to coronary sinus. **C** T2-weighted image in CMR. **D** T1 weighted late enhancement image after contrast in CMR

On admission, her chest was sore, and breathing was painful. The chest x-ray revealed a grossly enlarged cardiac silhouette prompting a suspicion of a cardiac tamponade (Fig. 1A). Transthoracic echocardiogram (TTE) revealed a large cystic mass within or adjacent to the pericardial sac that compressed the lateral wall of the normal-sized left ventricle (LV) with a preserved systolic function. A mild mitral valve insufficiency was noted. No pericardial fluid was detected. Contrast-enhanced computed tomography (CT) of the heart and coronaries confirmed an intrapericardial cystic tumor with a 14 cm diameter, which was located mostly in the left and caudal aspects of the heart (Fig. 1B). The arterial blood supply originated from distal branches of both the left circumflex (LCX) and right (RCA) coronary arteries. The venous drainage was to the coronary sinus. No calcifications, fat, or enhancement were found in the tumor.

In cardiac magnetic resonance (CMR) imaging, the pericardial tumor consisted of several septal hemorrhagic lacunae and vessels, which were hyperintense in both T1- and T2-weighted images. Figure 1C demonstrates the T2-weighted images in CMR. According to fat-suppressed images, there was no significant fat consistency in the tumor, and it did not enhance clearly by contrast (Fig. 1D).

To detect any malignant tumors in the chest or abdominal cavities, a whole-body CT was carried out. No malignancies were found thus excluding the possibility of cardiac metastasis. The patient had recent fractures in left costae 10–12 and left transverse processes of L1 and L2 vertebrae corresponding to the history of chest trauma.

Taken together, the imaging studies suggested that the tumor was either a hemangioma or an asymptomatic and slow-growing angiosarcoma. The absence of any calcifications and the lack of intense enhancement after contrast administration typical for hemangiomas, supported the preoperative diagnosis of an angiosarcoma.

**Fig. 2:**
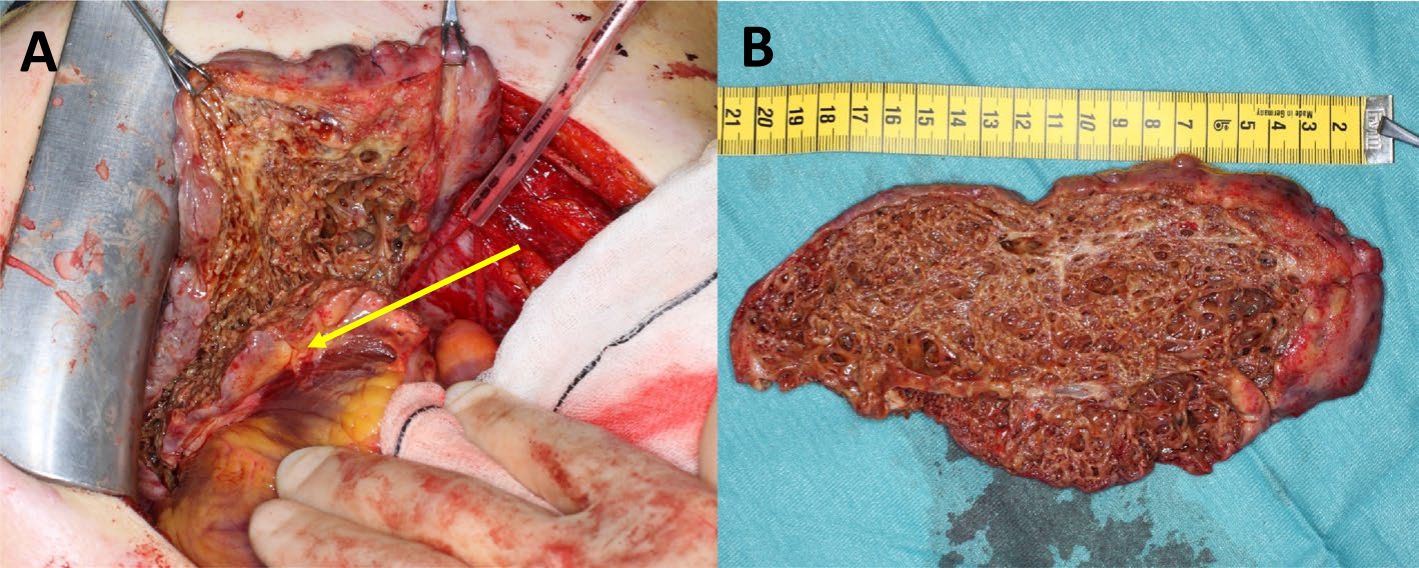
Tumor resection. **A** Posterolateral wall of the heart exposed. The tumor is partially resected and retracted. It infiltrates into the myocardium at the base of the heart (arrow). **B** The resection surface of the malformation showing the cavernous structure

A sternotomy with cardiopulmonary bypass and cardiac arrest was performed. The tumor originated with a large base both from the lateral and posterior wall of the LV infiltrating deep into the myocardium and close to the mitral valve annulus without a clear plane of dissection (Fig. 2A). Only partial resection could be performed (172 g of tissue, Fig. 2B).

**Fig. 3:**
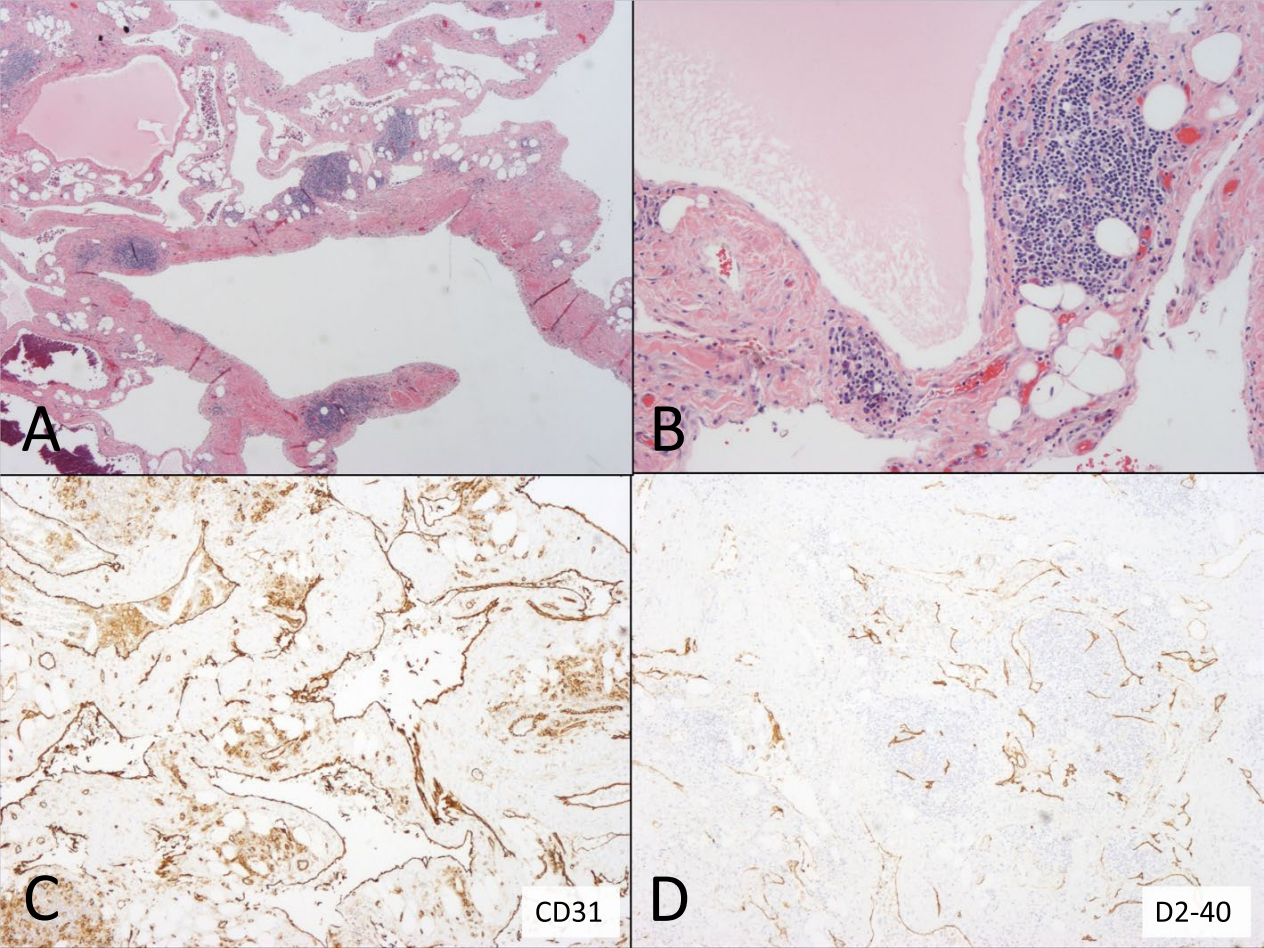
Histology of pericardial tumor. **A** and **B** Hematoxylin and eosin staining (original magnification × 20 and × 100). **C** and **D** Stainings for pan-endothelial marker CD31 and lymphatic endothelial marker podoplanin/D2-40 (original magnification × 40)

In histology, the tumor consisted of sponge-like tissue, with cystic spaces of 0.5–10 mm in diameter (Fig. 3A). Connective tissue septa contained paucicellular fibrous tissue, mature adipocytes, and aggregates of small lymphocytes (Fig. 3B). Cystic spaces were lined with non-atypical endothelial cells that were positive both for the pan-endothelial marker CD31 and the lymphatic endothelial marker podoplanin/D2-40 (Fig. 3C-D). No mitotic activity in the endothelium was observable. Cystic spaces contained either proteinaceous lymph, erythrocytes, or hemosiderophages derived from previous episodes of hemorrhage. Histopathological diagnosis was a cardiac lymphatic malformation with micro-and macrocystic components.

**Fig. 4:**
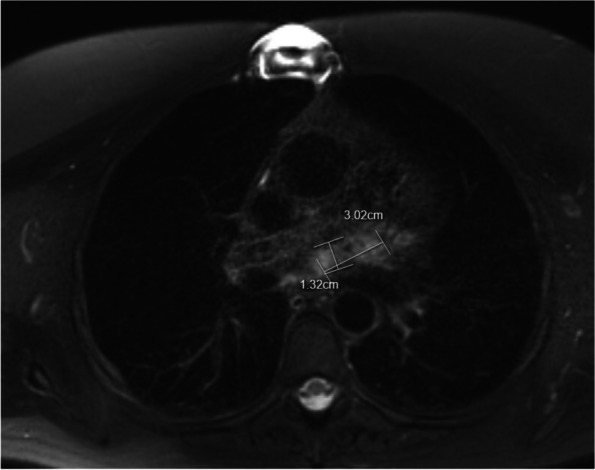
CMR Follow-up. CMR imaging of the tumor nine years after the surgery

The postoperative course was uncomplicated. At discharge, the maximum thickness of the residual tumor was 30 mm in TTE. The patient has been followed-up with decreasing intensity both with CMR and TTE. In the last CMR conducted nine years after the procedure, LV function was normal, and the maximum diameter of the residual tumor had regressed to 13 mm (Fig. 4). Latest follow-up performed by a cardiologist was 13 years after the operation; the patient had remained asymptomatic and used no medication. In TTE, LV size and function were normal, and the residual tumor had remained the same size. By June 2023, the patient had not contacted health care services outside the regular follow-up.

## Discussion and conclusions

Primary cardiac and pericardial tumors are very rare and mostly benign [1]. Mesothelial cysts are the most common pericardial primary tumors comprising majority of the cases, followed by teratomas, bronchogenic cysts, benign fibrous tumors, lymphatic malformations, and hyperplastic lymph nodes [1]. Mesotheliomas and sarcomas are the most frequently found types of malignant pericardial diseases [4]. Although rare, angiosarcomas can extend to pericardium or have a pericardial origin, and low-grade angiosarcomas can be difficult to differentiate from hemangiomas. Hemangiomas are rare benign cardiac tumors that may affect both pericardium and myocardium [1]. In our case the imaging studies suggested that the tumor was either a hemangioma or an angiosarcoma. The absence of any calcifications and the lack of intense enhancement after contrast administration typical for hemangiomas, supported the preoperative diagnosis of an angiosarcoma.

In our case histological studies confirmed the diagnosis of lymphatic malformation. Lymphatic malformations are a type of vascular malformation and can be divided to two major subtypes: micro- and macrocystic [5]. Majority of the lymphatic malformations are found in pediatric patients, although lymphatic malformations have been reported in adults as well [3]. Cardiac lymphatic malformations are very rare as lymphatic malformations are typically located in the head, neck, or axillary region [3]. The manifestation of symptoms depends on the location and the size of the tumor. In our case the patient had remained asymptomatic, and the tumor was found incidentally. Similarly, in previous reports some of the patients with cardiac lymphatic malformations had remained asymptomatic [2, 6, 7]. The most common symptoms related to cardiac lymphatic malformation were chest pain, dyspnea, respiratory distress, or palpitations [2, 8–11]. Surgical removal has been the typical treatment of choice. In some previous reports the tumors have been slightly smaller when compared to our case, and in some cases a complete removal has been possible [9, 11, 12]. In previous studies the results of surgical treatment have been good with no recurrence, but the follow-up time has ranged from only one month to two years [7, 9, 11, 12]. We report a significantly longer follow-up time when compared to most previous studies. Our case demonstrates that if a complete removal is not possible, a debulking procedure can yield a good long-term result. In case of recurrence a repeat debulking procedure could be considered.

## Data Availability

All data generated or analyzed during this study are included in this published article.

## Abbreviations

CMR: Cardiac magnetic resonance imaging
CT: Computed tomography
LCX: Left circumflex
LV: Left ventricle
RCA: Right coronary artery
TTE: Transthoracic echocardiogram
